# Physical Activity in Patients With Epilepsy: A Risk Factor or a Healthy Habit?

**DOI:** 10.7759/cureus.102363

**Published:** 2026-01-27

**Authors:** Katarzyna Zakrys, Mikolaj Zakrys, Szymon Stupnicki, Mateusz Szot, Aleksandra Oparcik, Jakub Tarczykowski, Natalia Kwasniewska

**Affiliations:** 1 General Practice, Uniwersytecki Szpital Kliniczny w Poznaniu, Poznań, POL; 2 General Practice, Szpital Wojewódzki w Poznaniu, Poznań, POL; 3 General Practice, Wojewódzki Szpital Wielospecjalistyczny im. Dr. Jana Jonstona w Lesznie, Leszno, POL; 4 General Practice, Centrum Medyczne Hipolit Cegielski Poznan (HCP) Medical Center, Poznań, POL; 5 General Practice, Wojskowy Instytut Medyczny-Państwowy Instytut Badawczy, Warszawa, POL

**Keywords:** eeg, epilepsy, exercise-induced seizures, physical activity, post-traumatic seizures, risk assessment, seizures, sport discipline

## Abstract

The approach to physical activity in patients with epilepsy has substantially changed over the last decade. Despite multiple positive effects of physical activity on general health and well-being, patients with epilepsy have long been advised not to engage in sports activities. Recent studies have led physicians to formulate updated recommendations and to encourage patients to remain active. It has been demonstrated that sport does not increase seizure prevalence, and the rate of sport-induced injuries in people with epilepsy is comparable to that of the general population. Additionally, physical activity modulates brain plasticity through a number of mechanisms, including the effect of brain-derived neurotrophic factor (BDNF), gamma-aminobutyric acid (GABA)/glutamate balance, and maintaining long-term potentiation states in synapses. The International League Against Epilepsy (ILAE) classifies sports into three categories according to the potential risk of injury in the event of a seizure. While most activities fall into low- or moderate-risk groups, high-risk sports include aviation, climbing, diving, horse racing, motor sports, parachuting, rodeo, scuba diving, ski jumping, solitary sailing, surfing, and windsurfing. Qualification for sports participation requires individual assessments of predispositions, seizure type and frequency, reaction to specific sports disciplines, respiratory function, and adjustment of hydration and nutrition. The intensity of training should be increased gradually to avoid triggering factors, such as hyperventilation, alkalosis, and hyperthermia. Seizure occurrence differs between aerobic and anaerobic sports, which is another aspect that needs to be included. Exercise electroencephalographic (EEG) and ambulatory EEG monitoring should be taken into account in patients with exercise-induced seizures to optimize their training plan. Despite the evolving recommendations, it is difficult to formulate universal recommendations for everyone. Each patient with epilepsy should undergo an individual qualification process and be appropriately monitored.

## Introduction and background

Epilepsy is a neurological condition affecting approximately 0.76% of people worldwide, characterized by recurrent seizures [1]. Clinical research and scientific reviews show that 25-40% of them participate in different sport disciplines, which is a lower proportion than the general population [2,3]. Despite multiple positive effects of physical activity on general health and well-being, patients with epilepsy (PWEs) have long been advised against exercise due to concerns about triggering seizures [4]. Currently, a different approach has been adopted. Based on available research, sport does not negatively affect seizure prevalence and sport-induced injuries are not more common in PWEs than in the general population [4,5]. In fact, it may help to control seizure frequency and lead to broader psychosocial and health benefits. Nevertheless, aspects that need to be considered before undertaking the physical activity by PWEs are the type of seizures, their frequency, seizure precipitating factors, the type of sport and the willingness to take risks connected with sports participation [4]. This review examines the evidence on physical activity in epilepsy, highlighting benefits, risks, and safe participation guidelines.

Methodology

This study is a narrative review of the recent literature on the benefits, risks, and considerations about physical activity in patients with epilepsy (PWEs). The PubMed and Google Scholar databases were searched in November 2025. Articles in English published from 2015 onward were included in this review. Older articles were cited in order to define terms or show different approaches to the topic through years. During the literature search, the following keywords and their combinations were used: “epilepsy,” “physical activity,” “sport,” “athletes,” “physical exercise,” and “exercise-induced seizures.” Following a comprehensive literature search, more than 1,000 results meeting the search criteria were identified. An initial screening of titles and abstracts for relevance was conducted, followed by full-text evaluation of articles deemed potentially eligible. Studies that did not refer to physical activity in epilepsy were excluded. We focused on systematic reviews and randomized controlled trials (RCTs), as they represent the highest level of evidence. However, some case reports were included when we reported on aspects with limited data. 

This review was not conducted as a systematic or scoping review and therefore does not fully comply with the Preferred Reporting Items for Systematic Reviews and Meta-Analyses (PRISMA) or PRISMA-Scoping Reviews (ScR) guidelines. As this is a narrative review, no new statistical analyses were performed. All statistical data and quantitative outcomes presented are based exclusively on the results reported in the included studies.

## Review

Benefits of physical activity in epilepsy

Engagement in regular physical activity has been shown to confer significant benefits for general health and longevity, including the modifying of synaptic plasticity, reducing the frequency of seizures and neuroinflammation, and improving memory and cognitive function, which is especially important in PWEs. The physiological effects of training are mediated by different molecules such as neurotrophic factors, signal transduction proteins, transcription factors, and synaptic proteins [6]. 

Synaptic plasticity is a process of modulating the effectiveness of synaptic transmission in response to the level of input activity. The examples of change in synaptic activity are long-term depression (LTD) and long-term potentiation (LTP), which are induced differently in different parts of the brain. LTP is known to be connected with strengthening memory, while LTD is responsible for memory loss [6]. Numerous studies on the effect of physical activity on cognitive function suggest the beneficial influence of training stimuli on LTP duration. The study on animal models showed the accelerated rate of learning-dependent LTP state arising in the hippocampus due to physical exercise by the improved conductance level, timing, and probability of opening of N-methyl-D-aspartate (NMDA) receptor channels [7]. In another animal research, the proposed markers of increased synaptic conductivity state were the population spike (PS) amplitude and the field excitatory post-synaptic potentials (fEPSP). After running for 10 days, both measured values increased suggesting augmented synaptic efficacy [8]. 

Brain-derived neurotrophic factor (BDNF) is a neurotrophic protein that binds to its receptor, tropomyosin receptor kinase B (TrkB), which is widely expressed in the brain. It is present mostly in the hippocampus and is responsible for axonal regeneration, cognitive modulation, neuroplasticity, synaptogenesis, and angiogenesis [9]. One study conducted on male Wistar rats with pilocarpine-induced epilepsy investigated the effects of aerobic exercise on the BDNF-TrkB signaling pathway. The results showed that physical exercise increased the BDNF level, normalized the expression of TrkB genes and activated the BDNF-TrkB pathway, which can result in increased neuronal plasticity and facilitated cognitive function [10]. However, the interindividual variability and methodological limitations should be taken into account to avoid overgeneralization. These findings were confirmed in other animal models, in which regular training reduced the frequency of seizures and regulated the disturbed signaling pathways in the brain [11]. Similar conclusions come from a huge meta-analysis studying the effects of physical activity on BDNF in neurodegenerative diseases [12].

The rate of BDNF release is altered by multiple variables such as the type of exercise, time of training, and individual predispositions. The most efficient in modulating BDNF type of activity seems to be aerobic training, especially high-intensity interval training (HIIT). In the meta-analysis of seven databases, HIIT resulted in 35% increase in BDNF release, compared with 20% increase in response to long-term moderate exercise [13]. Additionally, it was discovered that the effect of resistance training on serum BDNF is significantly enhanced by aerobic activity [14]. According to the duration of training, the optimal time range resulting in the most efficient BDNF release has been measured as 30-60 minutes. After prolonged exercise, exceeding 90 minutes, the positive effect is neutralized by increasing cortisol concentration that suppresses BDNF expression [15]. 

Taking into account the influence of physical activity on neuronal excitability, it is important to study the mechanisms of epileptic discharges activated or inhibited by sport. The exercise indirectly influences the gamma-aminobutyric acid (GABA)/glutamate neurotransmitter balance, which may be both beneficial or harmful for PWEs, depending on the sport intensity and MRI parameters used in particular studies. Physical activity intensifies the expression of GABA_A receptors and enzymes used to synthesise GABA neurotransmitters, which decreases the seizure occurrence and lengthens the latency period in animal models [16]. Human studies have shown that physical training increases the non-oxidative consumption of carbohydrates in the brain, facilitating the de novo synthesis of amino acid neurotransmitters, such as GABA and glutamate. This sudden increase in some vulnerable individuals may temporarily decrease the seizure threshold and intensify seizures, but this effect is acute and dependent on the intensity of training [17]. Further research has to be done on how these acute effects contribute to long-term training and seizure control.

Risks and considerations

The International League Against Epilepsy (ILAE) prepared a consensus paper, which sums up the recommendations about participation of PWEs in sport activities and divides sport disciplines into three categories depending on the risk of injury: low, moderate, and high risk. According to this paper, the disciplines generating the highest risk are: aviation, climbing, diving, horse racing, motor sports, parachuting, rodeo, scuba diving, ski jumping, solitary sailing, surfing, and windsurfing. The risk is significant if the seizure occurs during the activity. Most of these activities require high altitudes or water environments and are generally considered as extreme, even for the healthy population. The chance of seizure occurrence during any of these performances depends on personal factors and has to be evaluated individually. Another two groups contain safe or moderately safe disciplines connected with a low risk of injury for the patient or people surrounding individuals, even during the seizure. The safest sports turned out to be: athletics, bowling, sports on the ground (baseball, basketball, cricket, field hockey, football, rugby, volleyball, etc.), cross-country skiing, curling, dancing, golf, and racquet sports [4]. 

When estimating the risk of physical activity, it is also important to take into account the most common mechanisms of head trauma that can result in the induction of seizures. The data obtained from the population-based epidemiologic study on the incidence of sport-related traumatic brain injury (TBI) and risk factors of severity in South Carolina suggest that the most common mechanisms by which TBI occurs are being kicked in football, followed by a fall injury. Severe TBI was associated with off-road vehicular sport, repeated head trauma, injuries during horse riding, and falls in different activities [18]. These events essentially increase the prevalence of epilepsy in the healthy population and worsen the symptoms in PWEs who are especially exposed to such accidents during seizures.

Exercise-Induced Seizures

Exercise-induced seizures are a type of reflex epilepsy and can be described as the epileptiform discharges occurring in the brain during or soon after the specific type of physical activity, which acts as the complex triggering factor. The symptoms include speech arrest and rapid limb or head movements. Based on the data obtained from case reports, it often manifests as focal seizures, particularly from the left temporal lobe and is induced by repetitive movements. Seizures induced by the exercise can happen both in generalized and focal epilepsy, but it seems to be most commonly related to the focal origin. However, further research on larger groups of people should be conducted [19,20]. 

There are only a handful of cases of exercise-induced seizures described in the literature. Although in theory exercise may trigger epileptiform activity in the brain, this occurs in a small group of PWEs, only in some episodes and seems to be an exception rather than a rule [21,22]. Based on the available data, 2-10% of PWEs may experience exercise-induced seizures [23,24]. Such a wide prevalence range may result from the heterogeneity of the studied populations and methodological differences. In spite of this, most publications suggest sport to be a potential candidate for non-pharmacological seizure treatment rather than a trigger [25-28]. 

Potential neurophysiological mechanisms through which physical activity may lower the seizure threshold are the autonomic and metabolic changes, such as the activation of the sympathetic nervous system, changes in pH value, local hypoxemia, hypoglycemia, hyperhydration, hyponatremia, muscle hyperthermia, fatigue, stress, repeated head trauma in contact sports, and excessive aerobic training [25,29]. Additionally, in some PWEs who are particularly sensitive to carbon dioxide changes, hyperventilation during exercise may induce seizures [19]. Some reports suggest that exercise may alter the antiepileptic drugs (AEDs) concentration by increasing the hepatic enzyme metabolism [30-33].

Firstly, based on the literature, it seems that the influence of metabolic disturbances, such as hyperthermia, hypoxia, hyperhydration, hypoglycemia, and hyponatremia, mostly contributes to the acute symptomatic seizures rather than the reflex epilepsy [30]. Secondly, hyperventilation, known as a trigger factor at rest, in response to sport, and increasing acidosis can even suppress seizures by preventing hypercapnia [30,31]. Finally, exercise can increase the metabolism of the old generation AEDs through faster drug clearance and protein binding sites competition, leading to insufficient blood concentration. However, the literature lacks robust evidence for this theory, suggesting only slight variations in the metabolism of phenytoin, phenobarbital, and valproic acid in response to exercise. Nevertheless, the difference is not statistically significant and does not influence seizure occurrence. Since a small sample was tested, the results may not be representative and should be considered with caution [33]. 

The susceptibility to triggering factors depends on the type of epilepsy. Some specific epileptic syndromes are more prone to particular triggering stimuli, but the relationship with activity is ambiguous. The case reports published so far document exercise-induced seizures both in generalized tonic-clonic epilepsy and focal epilepsies of frontal and temporal lobe origin [19,34-37]. In some cases, patients presented with a high frequency of seizures or seizures occurring only during physical activity. Researchers have suggested that this might be a temporal lobe reflex epilepsy and that the temporal lobe, in comparison to other cortical areas, might be extremely sensitive to generate epileptiform impulses in response to exercise stimuli [19,22,37]. 

Neurophysiological Differences Between Idiopathic and Post-traumatic Epilepsy in Contact Athletes

Sport-related concussions affect 4.17 per 10,000 high school athletes performing any sport discipline, with football being considered to be the sport with the highest risk [38]. Adolescents were highlighted as an important group of people due to their high rates of post-traumatic epilepsy and the data availability. The injury and athlete exposure data were provided by the trainers to the National High School Sports-Related Injury Surveillance Study. Most concussions do not result in serious long-term consequences. However, some of them lead to neurological disorders, such as post-traumatic epilepsy [39]. Post-traumatic epilepsy is diagnosed after the first unprovoked seizure that occurs after a potentially epileptogenic brain insult, which is, among others, traumatic brain injury (TBI) [40]. It accounts for 10-20% of all cases of epilepsy in the general population [41]. The knowledge about the progression of neural injury and the development of late-onset seizures is still limited. 

The literature lacks reliable diagnostic or prognostic biomarkers of post-traumatic concussion, which makes it difficult to implement an optimal therapy. Nevertheless, there are some well-known differences between idiopathic and post-traumatic epilepsy that can be expressed through electroencephalographic (EEG) patterns, neuroimaging, the mechanisms of epileptiform activity, treatment, monitoring, and prognosis. 

The EEG features in post-traumatic epilepsy comprise localized, focal changes, typical for the injury of a particular part of the brain, such as focal slowing in the lesion area and, sometimes, a disturbed EEG background, depending on the severity and localization of injury [42]. In contrast, in idiopathic generalized epilepsy, the EEG pattern shows generalized spike or polyspike, slow-wave discharges at 35 Hz, normal background cortical rhythms, and a relatively high occurrence of photosensitivity. They often worsen during non-rapid eye movement (NREM) sleep and before awakening [43]. 

Evidence from magnetic resonance imaging, sensitive to detecting mesial temporal sclerosis, shows a significant correlation between the EEG pattern and the image in post-traumatic epilepsy. After the injury, deviations are focal and demonstrate structural damage and local microstructural changes (diffusion tensor imaging (DTI)) corresponding to the site of injury, which include microgliosis, microhemosiderin, and atrophy [44,45]. In idiopathic generalized epilepsy, neuroimaging does not correlate so precisely with the EEG results, showing more subtle, diffuse network changes without one focus [46,47].

The mechanisms of epileptiform discharges in post-traumatic seizures are multifactorial and complementary. After the injury, the neuroinflammatory response is activated, recruiting microglia, astrocytes, and cytokines, which promote neuronal excitability. Additionally, the blood-brain barrier damage leads to the influx of plasma proteins that influence the astrocyte signaling and neurotransmission disorders. The injury causes electrolyte channels and GABA/glutamate receptors imbalance in favor of excessive excitability, which further leads to neuronal death. After that, new astrocyte over-exciting connections are created. Oxidative stress and mitochondrial dysfunction arising as a consequence of injury further facilitate neuronal damage and improper synaptic reconstructions [48-50]. However, it was observed that genetic differences influence the individual's susceptibility to inflammatory response, tissue repair, and epileptogenesis, which explains why not everyone after the brain damage develops post-traumatic epilepsy [51]. In idiopathic epilepsy, the mechanisms concentrate on genetically determined ion channels and receptor abnormalities, leading to impaired thalamocortical excitability and network synchronization [52].

The optimal treatment of post-traumatic epilepsy depends on the severity of injury and time from TBI to first seizure and can include pharmacological and surgical therapy [53]. Most concussions do not result in epilepsy, so the prognosis is good and routine chronic use of antiepileptic drugs (AEDs) is not recommended. AEDs are usually used in the first seven days after severe TBI as a method of prevention [54,55]. The most researched AED in this indication is phenytoin, but levetiracetam has recently gained growing popularity [56]. Long-term AED therapy is recommended after the first late seizure due to the high risk of recurrence [57]. The types of AED used in post-traumatic epilepsy do not differ from those used in other types of epilepsy; however, response to them is often insufficient to effectively control seizures [58,59]. Therefore, some patients require surgical inactivation of epileptiform foci. Other options for patients not qualified for surgery are vagus nerve stimulation and responsive neurostimulation [60,61]. 

Recommendations for safe participation

Together with evolving therapeutic approaches, different recommendations for PWEs regarding participation in sport activities have been proposed. Firstly, each PWE should have an individual training plan focused on their own needs and taking into account the respiratory function, seizure type and frequency, as well as the reaction to a particular sport discipline. Such individualized assessment could be coordinated by a team of specialists, including neurologists, dietitians, and trainers. The intensity of training should be increased gradually in order to improve VO2 max and avoid hypoxia. Monitoring of glucose, sodium, and potassium levels, together with optimal hydration and avoiding hyperthermia, can help to control seizures and plan the activity. Another important aspect is an appropriately adapted nutrition and hydration plan before, during, and after the exercise. Ambulatory EEG should be considered in individuals with a high probability of exercise-induced seizures.

The Role of Exercise and Ambulatory EEG Monitoring in Sports Qualification

Exercise EEG is a tool used in order to record the bioelectrical changes during physical exertion. The electrodes are attached to the patient's head during the exercise on the treadmill or ergometer. At the same time, the signals from the electrodes and the patient's physiological parameters are recorded. Considering the qualification of PWEs to sport activities, the doctor can test the patient's reaction to stimuli in safe conditions. The exercise can potentially induce epileptiform discharges by the induction of hyperventilation, hyperthermia, hypoglycemia, hyponatremia, hypoxia, fatigue, stress of competition, increase of body temperature, and heart rate fluctuations [62]. That's why the seizure occurrence differs between aerobic and anaerobic sports.

Exercise EEG is successfully used in the risk assessment in the context of clinical recommendations. The systematic review of 42 papers in which exercise EEG was used to evaluate the risk of seizure occurrence revealed that in most cases, exercises did not increase the rate of seizures. Ten of these studies were classified as evidence level 3, 27 as evidence level 2-, 2 as evidence level 2+, and 3 as evidence level 1-. This resulted in the conclusion that sport shouldn’t be forbidden for PWEs and they should rather be encouraged to do so [5]. The study conducted on 26 children with intractable partial and generalized epilepsy provided proof that physical activity can both indicate seizures, but even more often alleviate them. The results showed that in 20 out of 26 tested by exercise EEG children, the epileptiform discharges decreased during the activity [63]. The use of exercise EEG in the case series of 10 patients with exercise-induced seizures allowed researchers to confirm the diagnosis and led to conclusions that exercise might be an underrecognized form of reflex epilepsy. These findings were crucial for giving advice about the avoidance of known trigger factors, which is a part of the management of these patients [19]. 

Continuous neurophysiological monitoring (ambulatory EEG/outpatient EEG) is another way to record the epileptic discharges during sport activities. To date, most literature sources describe the use of ambulatory EEG without video monitoring, which is the basis of diagnosing in epilepsy centers. However, recent technological development made it possible to do it without the need of hospitalization [64]. This wearable outpatient technique is especially useful for athletes who need real-time monitoring during the performance. Apart from recording epileptiform activity, it enables monitoring of an athlete’s fatigue and concentration, improving the strategy and optimizing their training and rehabilitation to prevent injuries. The neurophysiological markers of concentration are the decreased theta bands in frontal regions and increased beta and sensorimotor rhythm (SMR) activity, as well as a stable alpha rhythm with clear alpha blocking during task performance. Fatigue can be identified by the increase in theta activity and shift of the alpha peak frequency toward lower frequencies [65,66].

## Conclusions

In recent years, the attitude to participation of PWEs in sport activities has substantially changed. A sedentary lifestyle is no longer recommended, and the benefits of physical activity are considered to exceed the potential risk. Nevertheless, there are a few important steps that need to be followed by the athlete (either professional or amateur) in the context of safe training and competition participation. These include an appropriate diet, electrolyte monitoring, and individual assessment coordinated by specialists. Ambulatory EEG should be considered in individuals with a high probability of exercise-induced seizures. Epilepsy is a chronic disease, and despite the greatest desire to take part in all activities without limitations, there are still some high-risk sports that can lead to serious consequences. Sport disciplines that are not recommended include high-altitude and water sports, as well as extreme sports such as aviation, climbing, diving, horse racing, motor sports, parachuting, rodeo, scuba diving, ski jumping, solitary sailing, surfing, and windsurfing. Therefore, notwithstanding ongoing advances and the continuously evolving therapeutic approaches, individual patient assessment remains essential, with careful consideration of potential risks and benefits. Recommendations should be updated regularly as the new data become available.
